# *Agrobacterium*-mediated genetic transformation of yam (*Dioscorea rotundata*): an important tool for functional study of genes and crop improvement

**DOI:** 10.3389/fpls.2014.00463

**Published:** 2014-09-15

**Authors:** Evans Nyaboga, Jaindra N. Tripathi, Rajesh Manoharan, Leena Tripathi

**Affiliations:** International Institute of Tropical AgricultureNairobi, Kenya

**Keywords:** *Dioscorea rotundata*, *Agrobacterium*-mediated transformation, axillary buds, selection marker gene, reporter gene

## Abstract

Although genetic transformation of clonally propagated crops has been widely studied as a tool for crop improvement and as a vital part of the development of functional genomics resources, there has been no report of any existing *Agrobacterium*-mediated transformation of yam (*Dioscorea* spp.) with evidence of stable integration of T-DNA. Yam is an important crop in the tropics and subtropics providing food security and income to over 300 million people. However, yam production remains constrained by increasing levels of field and storage pests and diseases. A major constraint to the development of biotechnological approaches for yam improvement has been the lack of an efficient and robust transformation and regeneration system. In this study, we developed an *Agrobacterium*-mediated transformation of *Dioscorea rotundata* using axillary buds as explants. Two cultivars of *D. rotundata* were transformed using *Agrobacterium tumefaciens* harboring the binary vectors containing selectable marker and reporter genes. After selection with appropriate concentrations of antibiotic, shoots were developed on shoot induction and elongation medium. The elongated antibiotic-resistant shoots were subsequently rooted on medium supplemented with selection agent. Successful transformation was confirmed by polymerase chain reaction, Southern blot analysis, and reporter genes assay. Expression of *gusA* gene in transgenic plants was also verified by reverse transcription polymerase chain reaction analysis. Transformation efficiency varied from 9.4 to 18.2% depending on the cultivars, selectable marker genes, and the *Agrobacterium* strain used for transformation. It took 3–4 months from *Agro*-infection to regeneration of complete transgenic plant. Here we report an efficient, fast and reproducible protocol for *Agrobacterium*-mediated transformation of *D. rotundata* using axillary buds as explants, which provides a useful platform for future genetic engineering studies in this economically important crop.

## INTRODUCTION

Yam (*Dioscorea* spp.) is an economically important food crop in many tropical countries especially in West Africa, South Asia, and the Caribbean. It is the second most important root and tuber crop in the world after cassava in terms of production (Jova et al., 2005; Adegbite et al., 2006). Yam tubers are nutritionally rich and a major source of dietary fiber, carbohydrates, vitamin C, and essential minerals (Charles et al., 2005; Polycarp et al., 2012). In addition, they are also known for their secondary metabolites (steroidal saponins, diterpenoids, and alkaloids) which have been exploited for pharmaceutical products (Mignouna et al., 2008). There are 600 *Dioscorea* species, however, only 10 of about 90 edible species are regularly cultivated for food. *Dioscorea rotundata* and *D. cayenensis* (both known as Guinea yam) are the most popular and economically important yams in West and Central Africa, where they are indigenous (Mignouna et al., 2003; Adegbite et al., 2006; Quain et al., 2011), while *D. alata* (referred to as water or greater yam) is the most widely distributed species globally. The consumer demand for yam is very high in sub-Saharan Africa, but the yam production is declining in this region due to factors including diseases and pests, high costs of planting material, and decreasing soil fertility.

Diseases caused by viruses, fungi and bacteria and nematode pests either singly or in combination are responsible for yield losses (Nwankiti and Arene, 1978; Onwueme, 1978; Ng, 1992; Hughes et al., 1997). Nematodes are of particular concern because, apart from causing significant reduction in tuber yield and quality, they facilitate fungal and bacterial attacks. A major economic pest of yam is *Scutellonema bradys*, known as the yam nematode and causal agent of dry rot. This nematode occurs mostly in West Africa, where yam is its principal host, but is also recorded on yams from parts of South and Central America and Asia (Bridge et al., 2005). The nematode affects all the main cultivated yam species and cultivars in West Africa, mainly on mature tubers and during storage (Kwoseh, 2000; Coyne et al., 2006). Plant parasitic nematodes damage is also a critical factor in tuber quality reduction and yield loss in yam both in the field and storage (Adegbite et al., 2006). Yam nematodes reproduce and build up large populations in stored tubers causing severe damage and facilitating fungal and bacterial attacks that cause anthracnose disease, dry rot, soft rot, and wet rot.

The most important field pathogen of yam is the foliar anthracnose-causing fungus *Colletotrichum gloeosporioides*, which is a major threat to yam cultivation, in all yam-producing areas (Abang et al., 2002). The disease causes leaf necrosis and shoots dieback of yams, thus reducing the photosynthetic efficiency of the plant, which results in yield losses of over 90% in susceptible genotypes (Egesi et al., 2007). Yam viral diseases also constitute a major pathological problem in yam production in all growing regions of the world. The use of infected vegetative propagules and uncontrolled introductions of infected germplasm by farmers through porous land borders have resulted in the presence of yam viruses in all yam-growing areas of West Africa (Goudou-Urbino et al., 1996; Hughes et al., 1997). Viruses reported to infect yams in West Africa include *yam mosaic virus* (YMV), *yam mild mosaic virus* (YMMV), *D. dumetorum virus*, *D. alata bacilliform virus* (DaBV), *cucumber mosaic virus* (CMV), *D. mottle virus* (DMoV), and *D. sansibarensis virus* (DsBV; Seal and Muller, 2007). Yam viruses are of substantial economic importance not only because of yield losses they cause, but also due to the high cost of preventive measures (Degras, 1993).

Efforts have been made in the form of conventional breeding toward the development of pest and disease resistant and high yielding varieties. Transfer of desirable genes from the secondary gene pool of wild relatives to the cultivated primary gene pool remains difficult in many crops, including yams (Spillane and Gepts, 2001). Genetic improvement of yam through breeding programs face challenges due to constraints such as the long breeding cycle, dioecious, poor flowering nature, polyploidy, vegetative propagation, and heterozygous genetic background (Mignouna et al., 2008). Genetic engineering has emerged as a valuable alternative and complementary approach to improve crops including yam. Because of the difficulties surrounding conventional breeding of yam, the use of transgenic approaches to improve this crop is particularly compelling. However, the capacity to achieve successful genetic transformation depends largely on efficient plant regeneration systems. Regeneration systems of *D. rotundata* and *D. alata* have been established (Adeniyi et al., 2008; Tripathi et al., unpublished). Recently, direct shoot organogenesis was also reported on petiole explants of *D. rotundata*, *D. cayenensis,* and *D. alata* (Anike et al., 2012). These regeneration systems have not been evaluated for the amenability to transformation.

*Agrobacterium*-mediated transformation is the gene delivery system, which is most preferred by plant biotechnologists because of its easy accessibility, tendency to transfer low copies of DNA fragments carrying the genes of interest at higher efficiencies with lower cost and the transfer of very large DNA fragments with minimal rearrangement (Shibata and Liu, 2000; Gelvin, 2003). Therefore, plant transformation through *Agrobacterium*-mediated DNA transfer has become a favored approach for many crop species (Barampuram and Zhang, 2011). To date, there are only few reports of transient transformation of *D. alata* by particle bombardment using a reporter gene. Tör et al. (1993) successfully transformed cell suspension of *D. alata* by particle bombardment and found that the foreign gene (*gusA*) could be stably expressed in the transgenic cells; however, transgenic plants were not produced from transformed cells. Tör et al. (1998) further demonstrated that foreign genes could be delivered to protoplasts of *D. alata* using a polyethylene glycol-mediated uptake method. However, regeneration of transgenic plants was not reported. Quain et al. (2011) also reported transient transformation of *D. rotundata* using *Agrobacterium*; however, it cannot be applied for crop improvement since no transgenic plant was regenerated. As efficient transformation system for yam is currently not available, therefore, the main objective of this study was to establish an efficient *Agrobacterium*-mediated transformation system for *D. rotundata*.

## MATERIALS AND METHODS

### YAM CULTIVARS AND EXPLANT PREPARATION

Yam cultivars of Tropical *D. rotundata* (TDr) 2579 and 2436 were obtained as plantlets from *in vitro* germplasm collection at International Institute of Tropical Agriculture (IITA)-Ibadan, Nigeria. All the cultivars were maintained *in vitro* and multiplied as shoot cultures on yam basic medium (YBM) containing Murashige and Skoog medium (MS) salts and vitamins, 0.05 mg/l 6-Benzylaminopurine (BAP), 0.02 mg/l Naphthaleneacetic acid (NAA), 25 mg/l Ascorbic acid, 30 g/l sucrose, 2.4 g/l gelrite. The pH of the medium was adjusted to 5.8 prior to autoclaving. The cultures were incubated in growth room at 28^∘^C with 16/8 h photoperiod. The nodal explants (3–5 mm) containing axillary buds were excised from young *in vitro* shoots.

### SENSITIVITY OF AXILLARY BUD EXPLANTS TO ANTIBIOTICS

Prior to transformation experiments, the sensitivity tests to selective agents (hygromycin and kanamycin) were carried out in order to find an effective inhibitory concentration, which arrests the formation of shoot buds and shoots from nodal explants. The sensitivity to antibiotics was determined by culturing nodal explants having axillary buds on shoot bud induction medium (SBM; MS salts and vitamins, 1 mg/l BAP, 0.318 mg/l Copper sulfate, 20 g/l sucrose, 2.4 g/l gelrite) supplemented with different concentrations of hygromycin (0–15 mg/l) or kanamycin (0–250 mg/l). The cultures were transferred to a fresh medium containing the same level of antibiotic every 2 weeks and then scored for the frequency of regeneration after 8 weeks. The minimal inhibitory concentration of antibiotics was used in all the transformation experiments.

### *Agrobacterium* STRAINS AND BINARY VECTORS USED FOR TRANSFORMATION

**FIGURE 1:**
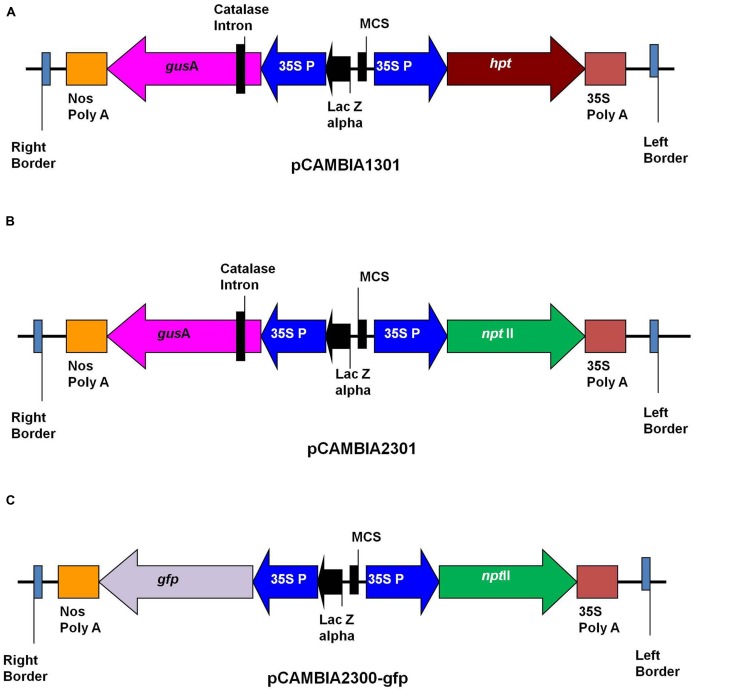
**Schematic representation of T-DNA of binary plasmids. (A)** pCAMBIA1301; **(B)** pCAMBIA2301; **(C)** pCAMBIA2300-gfp.

*Agrobacterium tumefaciens* strains LBA4404 and EHA105 were used in this study. The binary vectors pCAMBIA1301, pCAMBIA2301 (CAMBIA Company, Australia) and pCAMBIA2300-gfp were used for transformation (**Figure 1**). The pCAMBIA1301 contained hygromycin phosphotransferase(*hpt*) gene as selection marker, while pCAMBIA2301 and pCAMBIA2300-gfp contained neomycin phosphotransferase II (*npt*II) as selectable marker. Plasmids pCAMBIA1301 and pCAMBIA2301 contained the intron-containing *gusA* reporter gene, while plasmid pCAMBIA2300-gfp contained *gfp* as reporter gene. The binary vectors were transformed into *A. tumefaciens* strains LBA4404 and EHA105 by electroporation. Single colonies from Luria Bertani (LB) agar (10 g/l Tryptone, 5 g/l Yeast extract, 10 g/l Nacl, 15 g/l Agar, pH 7.5) plates containing kanamycin (50 mg/l), rifampicin (50 mg/l) and streptomycin (100 mg/l) were used to initiate 2 ml LB medium starter cultures. After 48 h shaking at 150 rpm at 28^∘^C, this suspension was used to inoculate a 20 ml LB medium containing the same antibiotics, and grown overnight on a shaking platform at 150 rpm to reach an OD_600_ of 1.0. Bacterial culture was centrifuged at 3500 rpm for 15 min and pellet was re-suspended in liquid SBM medium supplemented with 200 μM acetosyringone (Sigma Chemical Co.) and grown further for 2–3 h at 25^∘^C with shaking at 100 rpm. The optical density (OD_600_) of culture was checked and adjusted to 0.5. The bacterium suspension was used for transformation experiments.

### INOCULATION OF EXPLANTS WITH *A. tumefaciens* AND CO-CULTIVATION

The explants of *D. rotundata* were immersed in bacterial suspension and vacuum infiltrated for 5 min followed by gentle shaking at 45 rpm for 30 min at room temperature. After inoculation, explants were blotted on sterile paper towels and co-cultivated for 3 days under dark condition at 28^∘^C, in petri dishes containing SBM medium supplemented with 100 μM acetosyringone. Fifty explants were used in each experiment and transformation efficiency was compared with two *Agrobacterium* strains (LBA4404 and EHA105) and plasmids with different selection marker genes (*hpt* and *npt*II). Experiments were repeated twice.

### SELECTION AND REGENERATION OF TRANSGENIC PLANTS

Following co-cultivation, the explants were rinsed three to four times with liquid SBM medium supplemented with 500 mg/l carbenicillin and blotted dry on sterile filter paper and placed onto SBM supplemented with 250 mg/l carbenicillin for 1 week of recovery at 28^∘^C 16/8 h photoperiod. After 1 week of incubation, the explants were transferred to fresh SBM medium supplemented with 250 mg/l carbenicillin and 7.5 mg/l hygromycin or 100 mg/l kanamycin depending on the plasmid used and incubated for 14 days at 28^∘^C under 16/8 h photoperiod. This step was repeated twice with gradually increasing the antibiotic selection to 10 and 15 mg/l for hygromycin selection or 125 and 150 mg/l for kanamycin selection. The elongated shoots were separated and transferred to YBM containing 250 mg/l carbenicillin and 15 mg/l for hygromycin or 150 mg/l for kanamycin for 1 month for rooting. The well rooted plantlets were transferred to peat pellets, covered with transparent polythene bag and placed in a glasshouse at 28^∘^C. After 3–4 weeks, each peat pellet is fragmented and plantlets transferred into pots containing sterile soil and covered with plastic bags. When plants have reached 30–50 cm in height the plastic bags were opened to allow further growth. The putative transgenic plants regenerated on selective medium were subjected to β-glucuronidase (GUS) histochemical assay or fluorescent microscopy and molecular analysis.

### HISTOCHEMICAL GUS ASSAY

Transient and stable histochemical GUS assay was carried out in different tissues as described by Jefferson et al. (1987) with modifications. Tissues were immersed in a buffer containing 2 mM X-Gluc, 50 mM phosphate, 50 mM potassium ferrocyanide and 5% Trition X-100 at pH 7.0 and vacuum infiltered for 10 min, and then incubated overnight at 37^∘^C for 24 h. Tissues containing chlorophyll were repeatedly soaked in 95% ethanol until chlorophyll was removed. Transient expression of *gus*A gene was examined in *Agro*-infected explants after 3 days of co-cultivation, while stable expression of the reporter gene was analyzed in leaves, shoots and roots isolated from putative transgenic plants regenerated on selective medium.

### VISUALIZATION OF GFP FLUORESCENCE

Transient and stable GFP expression was analyzed using a Nikon SMZ1500 stereomicroscope with GFP-Plus fluorescence module. The images were recorded in TIFF format using a digital camera. All plants putatively transformed with pCAMBIA2300-gfp were tested for GFP expression.

### GENOMIC DNA ISOLATION AND PCR ANALYSIS OF TRANSGENIC LINES

Plant genomic DNA for polymerase chain reaction (PCR) was extracted from regenerated putative transgenic young leaves using a DNeasy kit (Qiagen, GmbH, Germany). Specific primers used for *gus*A were: forward 5′-TTTAACTATGCCGGGATCCATCGC-3′ and reverse 5′-CCAGTCGAGCATCTCTTCAGCGTA-3′. Specific primers for *hpt* were: forward 5′-CCACTATCGGCGAGTACTTCTACACAGC-3′ and 5′-GCCTGAACTCACCGCGACGTCTGTC-3′. PCR was conducted in a total volume of 20 μl, containing 100 ng template DNA, 2 μl 10 × buffer, 0.5 μl of 10 mM dNTP, 0.5 μl of 10 μM primers, 1 unit of Taq DNA polymerase (Qiagen, GmbH, Germany). The PCR conditions were: initial denaturation at 94^∘^C for 10 min, 35 cycles of denaturation at 94^∘^C for 15 s, annealing at 62^∘^C for 40 s for the *gus*A gene, 58^∘^C for 40 s for the *hpt* gene, and extension at 72^∘^C for 50 s, followed by final extension at 72^∘^C for 7 min and holding at 4^∘^C. The amplified PCR products were separated by electrophoresis on 0.8% (w/v) agarose gel stained with GelRedTM (Biotium) and visualized under a UV transilluminator and photographs were taken by the gel documentation system.

### RNA EXTRACTION AND RT-PCR ANALYSIS

Total RNA was extracted from 100 mg young leaf tissue of 10 transgenic lines and non-transgenic control plants using the RNeasy plant mini kit (Qiagen, GmbH, Hilden, Germany) and treated with DNase (RNeasy Plant Mini kit, Qiagen). The quantity and quality (A_260/230_ and A_260/280_) of total RNA were determined using the Nanodrop 2000. RNA was checked with PCR for absence of genomic DNA. Complementary DNA (cDNA) was synthesized using 1 μg of total RNA and reverse transcriptase of the Maxima H Minus First Strand cDNA synthesis kit with oligoDT primers (Thermo scientific). For reverse transcriptase polymerase chain reaction (RT-PCR), 2 μl of each cDNA synthesized was used. PCR cycling conditions included initial denaturation of 94^∘^C for 10 min, followed by 35 cycles of 94^∘^C for 15 s, 62^∘^C for 40 s and 72^∘^C for 50 s and final extension for 7 min. RT-PCR was performed with primers specific to the *gus*A gene as described above and housekeeping gene actin primers forward 5′- ACCGAAGCCCCTCTTAACCC-3′ and reverse 5′-GTATGGCTGACACCATCACC-3′. The amplified RT-PCR products were separated and visualized as described in the PCR section above.

### DOT BLOT AND SOUTHERN BLOT ANALYSIS

The integration of the transgene into the genome of yam was analyzed using dot blot and Southern hybridization. Genomic DNA for dot blot analysis was extracted from twelve PCR positive transgenic lines using a DNeasy kit (Qiagen, GmbH, Germany). About 200 ng of genomic DNA in triplicate for each transgenic line was denatured at 98^∘^C for 10 min, immediately chilled on ice for 5 min and immobilized onto a positively charged nylon membrane (Roche Applied Sciences, Mannheim, Germany) using a BIORAD Bio-Dot Microfiltration apparatus following the manufacturer’s protocols and recommendations. The DNA samples were fixed on the membrane by cross-linking in a STRATA-LINKTM UV cross-linker. A *gusA*-specific probe was labeled with DIG-dUTP using PCR DIG Probe Synthesis Kit (Roche Applied Sciences, Mannheim, Germany). Hybridization, stringency washes and detection was carried out using a DIG Luminescent Detection Kit for Nucleic Acids (Roche Diagnostics, UK) according to the manufacturer’s instructions.

For Southern blot analysis, genomic DNA was isolated from *in vitro* grown plants using cetyltrimethylammonium bromide (CTAB) method developed by Sharma et al. (2008) with modifications. The genomic DNA (20 μg) of transgenic lines and non-transgenic control plant was digested with *Hind*III (New England Biolabs, USA) for overnight at 37^∘^C. The plasmid DNA digested with *Hind*III was used as positive control. Restricted DNA was separated on a 0.8% (w/v) agarose gel at 40 V for 6 h and transferred to a positively charged nylon membrane (Roche Applied Sciences, Mannheim, Germany) by capillary transfer method and fixed by cross-linking in a STRATA-LINKTM UV cross-linker. Hybridization and detection was performed as described above.

### STATISTICAL ANALYSIS

Data were subjected to significance by analysis of variance (ANOVA) and mean separation by Duncan’s multiple range tests (DMRTs; *p* < 0.05) using SPSS 11.09 software for Windows.

## RESULTS AND DISCUSSION

### SELECTION OF SELECTABLE MARKER SUITABLE FOR YAM TRANSFORMATION

An effective selection strategy is very important for developing an efficient genetic transformation procedure. This can be achieved by the use of a selective agent which prevents non-transformed tissues from regenerating, while permitting the development of transformed cells into shoots without any lethality of the explant tissues (Song et al., 2012). The choice of selection agent depends on the plant nature and each plant species responds differently to the selection agent. The bacterial *npt*II and *hpt* are the most frequently used selectable marker gene used for generating transgenic plants. These enzymes detoxify aminoglycoside antibiotics by phosphorylation, thereby permitting cell growth and development of transformed plant cells into shoots in the presence of antibiotics. Therefore, optimization of the dose of selection pressure using hygromycin or kanamycin is important, as a suboptimal dose results in high frequency of escapes (Datta et al., 1990). On the other hand unnecessary high antibiotic doses not only kill untransformed tissues, but also inhibit growth of transformed cells, leading to delay in the regeneration process (Wilmink and Dons, 1993). Therefore, optimization of the aminoglycoside concentration was based on the minimal antibiotic concentration sufficient to prevent regeneration of untransformed tissues. The effective antibiotic concentration is another important factor for selection and regeneration of transgenic plant cells. Antibiotics decay in the plant tissue media due to various factors such as light, pH, temperature (Padilla and Burgos, 2010) as well as the antibiotic degradation in the vicinity of transgenic cells able to inactivate the antibiotic (Rosellini et al., 2007). Regular subculturing on fresh selection media as described in our study increases the effective inhibitory action of the antibiotic used.

**FIGURE 2:**
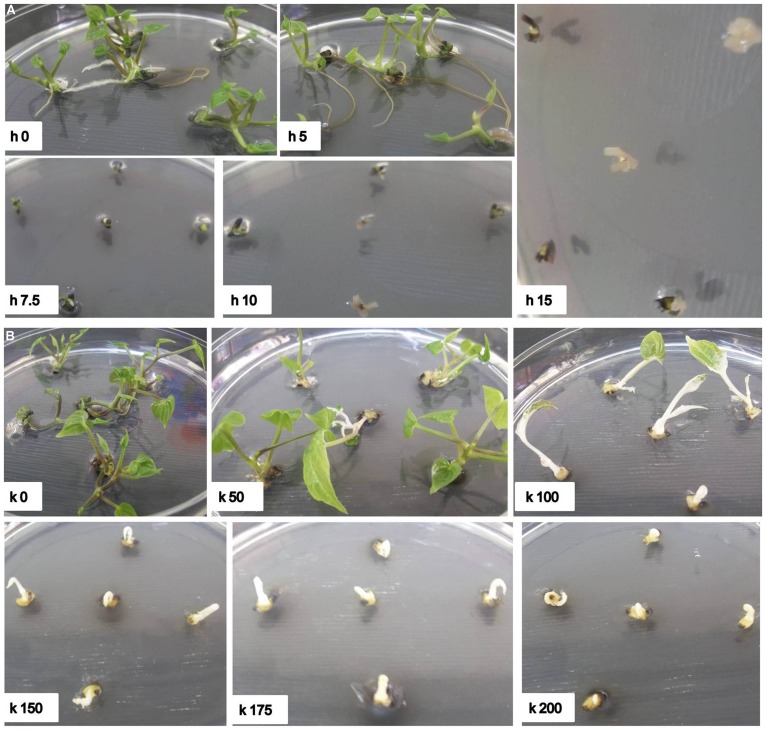
**Effect of hygromycin and kanamycin concentrations on regeneration of nodal explants of *D. rotundata*. (A)** h0–h15 represent culture conditions with different hygromycin concentrations where h refers to hygromycin and the number after h refers to different concentrations; **(B)** k0–k200 represent culture conditions with different kanamycin concentrations, where k refers to kanamycin and the number after k refers to different concentrations. The pictures were taken 30 days after culture in shoot elongation medium supplemented with antibiotics.

It has been reported that monocotyledonous plants are sensitive to hygromycin, but not to kanamycin (Hauptmann et al., 1988; Eady and Lister, 1998; Chin et al., 2007). Tör et al. (1993) also reported that the suspension cells of the *D. alata* showed a high tolerance to kanamycin and no growth inhibition even at a concentration of 500 ug/ml. However, our results indicated that shoot induction of *D. rotundata* is sensitive to both hygromycin and kanamycin (**Table 1**, **Figure 2**). Shoot bud induction and plant regeneration from axillary buds of nodal explants were completely inhibited on a medium containing 10 mg/l of hygromycin or 150 mg/l kanamycin. No study has been performed so far on the hygromycin-based selection for the transformation of yam. The effective inhibitory concentration of hygromycin and kanamycin determined through this study will assist with the design of selection conditions for both *hpt* and *npt*II gene-based plasmids in the future for effective transformation of yam and will also be useful to engineer yam with multiple T-DNA insertion.

**Table 1:** Effects of different concentrations of hygromycin and kanamycin on shoot bud induction and plant regeneration of *D. rotundata* using nodal explants.

Antibiotic	Concentration (mg/l)	Explant development/regeneration response
Hygromycin	0	Green shoots regenerated
	5	Green shoots regenerated
	7.5	Axillary bud induced but no shoot production
	10	No shoots regenerated
	15	No shoots regenerated and explants were bleached
Kanamycin	0	Green shoots regenerated
	50	Green shoots regenerated
	75	Green shoots regenerated with pale patches
	100	Shoots regenerated and turned completely white
	125	Albino shoots regenerated, arrested development
	150	No shoots regenerated
	200	No shoots regenerated
	250	No shoots regenerated

Efficient selection of transformed tissues was accomplished by increasing the selection pressure in a step wise manner from 7.5 to 15 mg/l and 100 to 150 mg/l for hygromycin and kanamycin, respectively. This process allows transformed explants to express effectively the antibiotic-resistance gene and initiate cell division, thus improving regeneration of explants to produce plants (Bull et al., 2009). Final selection on higher concentration of antibiotics also eliminates generation of false positive or escape plants. The use of low antibiotic concentrations in regeneration medium at early stages promotes transformed cell recovery, while a subsequent gradual increase of antibiotic concentration effectively eliminates non-transformed cells (Burgos and Alburquerque, 2003). Such pattern of selection was previously reported to be effective for cassava (Zhang and Puonti-Kaerlas, 2000), castor (Sujatha and Sailaja, 2005), dendrobium (Suwanaketchanatit et al., 2007), *Lotus corniculatus* (Nikolicć et al., 2007), grapevine (Fan et al., 2008), rapeseed (Liu et al., 2011), and spinach (Milojevicć et al., 2012) and also for jute, under kanamycin selection (Sarker et al., 2008).

### TRANSFORMATION, SELECTION, AND REGENERATION OF TRANSGENIC PLANTS

**FIGURE 3:**
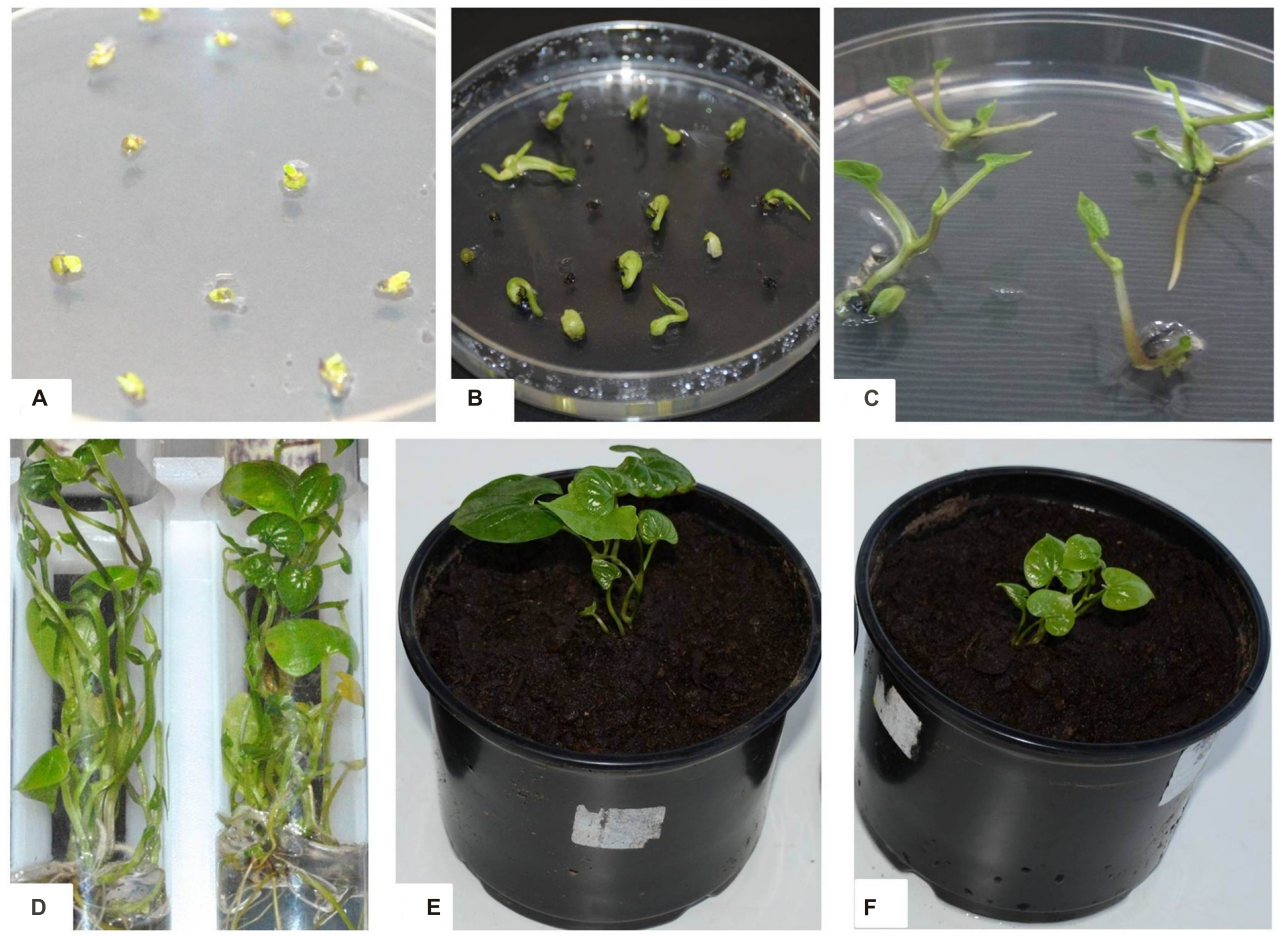
**Regeneration and transformation of *D. rotundata* cv. TDr 2436. (A)** Axillary bud induction from nodal explants after 1 week of culture on SBM; **(B)** shoot induction from nodal explants after 2 weeks culture on SBM; **(C)** proliferation of shoots within 8 weeks of culture on SBM; **(D)** rooting of elongated transformed shoot; **(E)** acclimatized transgenic plant maintained in glasshouse; **(F)** non-transgenic plant in soil in the glasshouse.

**FIGURE 4:**
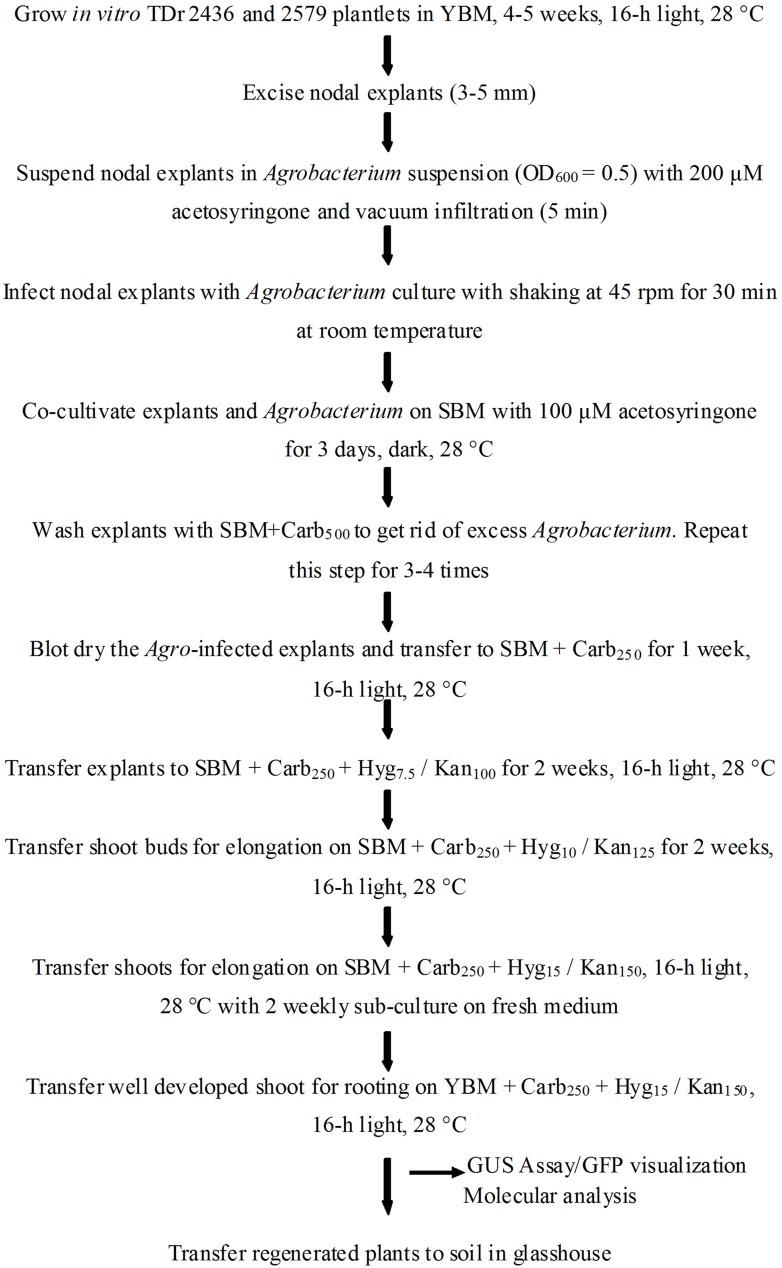
**Schematic diagram showing various steps of stable genetic transformation of *D. rotundata* using nodal explants**.

After co-cultivation, the explants were subjected to a resting period of 5–7 days in carbenicillin supplemented medium lacking selection agent to improve the regeneration of *Agro*-infected explants. It is reported that direct transfer to selective medium after co-cultivation could result in tissue necrosis of the explants (Khanna et al., 2007). *Agro*-infected nodal explants began to form axillary buds after 7 days on selective medium (**Figure 3**). The induced buds started producing shoots 4–6 weeks after *Agro-*infection on selective regeneration medium supplemented with gradual increase of antibiotics every 2 weeks (**Figure 3**). In 8–10 weeks some of the shoots elongated and turned green and other shoots turn white or chimeric. In this study, a clear difference was observed during kanamycin selection process of transformed (green) and non-transformed (bleached) developing shoot buds. In order to eliminate possible chimeric plants, the shoots produced were sub-cultured several times with the same level of selection pressure (15 mg/l or 150 mg/l kanamycin). After three subcultures, the chimeric shoots completely bleached and died while the transgenic shoots continued to survive and grow normally. All the shoots generated on selective medium produced roots when transferred onto YBM containing 15 mg/l hygromycin or 150 mg/l of kanamycin. In this rooting assay, only transformed shoots survived to rooting, whereas the escaping shoots did not produce roots. The putative transgenic plants generated were validated by PCR and proved to be transgenic by GUS assay and GFP fluorescence. A generalized scheme for stable genetic transformation protocol is shown in **Figure 4**.

The effect of cultivars, *Agrobacterium* strains and selection marker genes was examined on transformation efficiency using axillary buds as explants. Significant variation in transformation efficiency was observed among different cultivars based on *Agrobacterium* strains and antibiotic selection marker used (**Table 2**). We observed transformation efficiency of 9.4–18.2% depending on different transformation factors including the yam cultivars, *Agrobacterium* strains and antibiotic selection marker. Differences are known to exist between transformation efficiencies of different genotypes, expression vectors, selection marker genes, and the strain of *Agrobacterium* as well as the tissue culture conditions (Cheng et al., 2004). Among these factors, the cultivar of the explants is considered as a crucial one that can hardly be overcome or complemented through optimizing other external factors, for example by manipulating highly virulent strains (Hansen et al., 1994) or by optimizing plant culture conditions (Zuo et al., 2002).

**Table 2:** Effect of *Agrobacterium* strains, cultivars, selectable marker, and reporter genes on transformation efficiency of *D. rotundata.*

Plasmid construct	Cultivar	*Agrobacterium* strain	No. of regenerated plants on selective media	Transformation efficiency (%)
pCAMBIA1301	TDr2436	EHA105	9.4 ± 0.50^a^	18.2 ± 0.37^a^
		LBA4404	8.4 ± 0.24^ab^	16.2 ± 0.37^ab^
	TDr2579	EHA105	6.2 ± 0.37^cde^	12.8 ± 0.37^cd^
		LBA4404	7.6 ± 0.50^bc^	14.4 ± 0.24^c^
pCAMBIA2301	TDr2436	EHA105	7.4 ± 0.40^bc^	14.8 ± 0.37^c^
	TDr2579		4.8 ± 0.37^e^	10.2 ± 0.37^e^
pCAMBI2300-gfp	TDr2436	EHA105	7.2 ± 0.80^bcd^	14 ± 0.31^c^
	TDr2579		5.8 ± 0.37^de^	9.4 ± 0.24^e^

*The transformation experiments were repeated twice and 50 nodal explants were used for each transformation. Each value represents mean ± S.E. of two independent transformation experiments. Mean values followed by same letters within a column are not significantly different according to Duncan’s multiple range test (DMRT) at 5% level.*

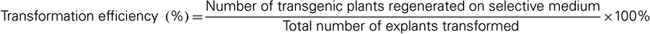

In the initial experiment we compared the effect of two *Agrobacterium* strains (EHA105 and LBA4404) harboring plasmid pCAMBIA1301 on the transformation efficiency of two cultivars of *D. rotundata*. No significant difference was observed in transformation efficiency due to various *Agrobacterium* strains used in the transformation (**Table 2**). However, there was a significant difference (*p* < 0.05) in transformation efficiency among the two cultivars transformed. The transformation efficiency was higher (16–18%) for cultivar TDr 2436 in comparison to cultivar 2579 (12–14%). As there is no significant effect of *Agrobacterium* strain used on transformation efficiencies, only one strain EHA105 was used for further studies. Our results suggest that different cultivars of the same species may differ remarkably in their susceptibility to *Agrobacterium* infection. The biochemical basis of these variations involves more complex mechanisms, as has been extensively reviewed by many authors (McCullen and Binns, 2006; Citovsky et al., 2007; Gelvin, 2010), that the transfer of DNA from *A. tumefaciens* to plant genome is a complex process involving a number of discrete, essential steps. The difference in the susceptibility of cultivars to *Agrobacterium* could be due to the presence of inhibitory metabolites to *Agrobacterium* sensory machinery (Liu and Nester, 2006; Maresh et al., 2006). Plant host defense response stimulated by *Agrobacterium* infection may be another factor influencing the susceptibility of plant cells to *Agrobacterium* (Ditt et al., 2005; Zipfel et al., 2006; Anand et al., 2008).

Selectable marker genes are required for establishment of efficient transformation in plants. In most cases, selection is based on antibiotic (kanamycin or hygromycin) or herbicide (phosphinothricin) resistance (Miki and McHugh, 2004). Selectable marker genes allow the plant cells that carry them to regenerate in media containing selective agents, while non-transformed cells die. The choice of a selectable marker gene depends on its efficiency, applicability to a wide range of plants, availability for researchers and its market acceptance (Kraus, 2010). In this study, we compared two selection marker genes (*hpt* and *npt*II) for transformation efficiency of two cultivars of *D. rotundata*. There was significant difference (*p* < 0.05) in transformation efficiency using *hpt* and *npt*II as selectable marker genes (**Table 2**). Although there was a significant difference between *hpt* and *npt*II selectable markers, our study demonstrates that hygromycin as well as kanamycin selection are efficient and can be used for the recovery of transgenic yam tissues and plants. No escape plants were obtained with any of the selection agents used. The *hpt*-hygromycin system has been reported to be more efficient than the *nptII*-kanamycin and the phosphinothricin acetyl transferase (PAT)-phosphinothricin systems (Song et al., 2012). However, the availability of multiple resistance gene/antibiotic selection systems that allow for efficient selection of transgenic yam is essential to generate multiple improved traits from independent T-DNA cassettes. From a regulatory perspective, *npt*II is particularly interesting since it is present in a large proportion of commercialized genetically modified crops (Miki and McHugh, 2004) and several independent studies have demonstrated its safe use in transgenic crops (Fuchs et al., 1993; Ramessar et al., 2007).

Other selection strategies that are free of antibiotic and/or herbicide-resistance genes can also be used to select transgenic plants. One of these strategies is the use of visual markers such as GUS (Jefferson et al., 1987) and green fluorescent protein (GFP; Prasher et al., 1992). In this study, we also compared two reporter genes (*gusA* and *gfp*) for the transformation efficiency of two cultivars of *D. rotundata*. There was no significant difference in transformation efficiency using *gusA* and *gfp* reporter genes for both cultivars (**Table 2**). Our results demonstrate that both *gusA* and *gfp* can be used as reporter genes for developing transgenic yam. The use of the *gusA* gene as a marker for transformation is effective and is widely applied in many species, including monocots such as rice (Wakasa et al., 2012) and orchard grass (Lee et al., 2006), and dicots such as common bean (Mukeshimana et al., 2013) and alfalfa (Duque et al., 2007). The presence of an intron in the *gusA* gene guards against false positives that may result from expression of the gene in *A. tumefaciens* (http://www.cambia.org). However, the GUS assay involves destruction of the tested tissues of transgenic explants; therefore, it should be performed at a later stage of the transformation study and presents a bottleneck for verification strategies in large-scale plant transformation protocols. GFP fluorescence visualization is also a useful tool for selecting transgenic yam plants. It has been widely used in the transformation of many plant species such as pepper (Jung et al., 2011), *Petunia hybrida* (Muβmann et al., 2011), and alfalfa (Duque et al., 2007). The fluorescent marker GFP is a highly versatile reporter gene, because the *gfp* gene expression can be monitored any time in living cells under a fluorescence microscope in a non-destructive manner (Chalfie et al., 1994). The same tissues may then be used for regeneration of stable transformants, which is not possible with other marker genes requiring destructive or toxic enzyme assays. Visual marker genes like *gfp* can even be used for yam transformation without using antibiotics or herbicides as the selection agents.

Complete transgenic plantlets ready for transfer to the greenhouse were produced within 3–4 months after *Agro*-infection. The rooted plants grew normally after transplanting to soil in the glasshouse (**Figure 3**) confirming that selection marker (*hpt* or *npt*II) or/and the reporter genes (*gus*A or *gfp*) does not have any apparent adverse effect on the normal development and morphology of the transgenic yam plants.

### GUS EXPRESSION AND VISUALIZATION OF GFP FLUORESCENCE IN TRANSGENIC PLANTS

**FIGURE 5:**
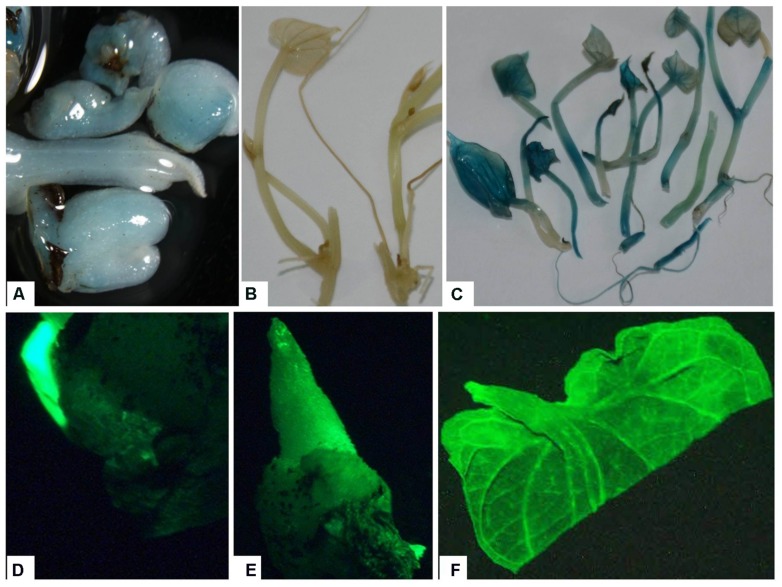
**Expression of reporter genes in tissues of putative transgenic plants of *D. rotundata.* (A)** Transient expression of *gusA* gene in emerging axillary buds 1 week after *Agro*-infection; **(B)** control non-transgenic plantlets; **(C)** stable expression of the *gusA* gene in transgenic plantlets; **(D)** transient expression of *gfp* gene in emerging axillary buds 3 days after *Agro*-infection; **(E)** gene expression in transgenic buds produced ∼1–2 weeks after *Agro*-infection; **(F)** leaf of transgenic plantlets viewed under UV light using GFP filter.

The use of both *gfp* and *gus*A genes as visual markers provides a useful method for confirming putative transformed plants (Padilla et al., 2006; Duque et al., 2007). The putative transgenic lines were verified by GUS histochemical assay or GFP fluorescence at different levels of plant development. The GUS staining was observed in nodal explants and emerging axillary buds indicating successful *Agro*-infection and transient expression of *gus*A gene (**Figure 5**). Transgenic plants displayed intense blue coloration in the leaf, stem, root, in contrast with non-transformed plant tissues, indicating the stable integration of the *gus*A gene into the genome and its expression (**Figure 5**).

In this study, we examined explants for GFP fluorescence at different stages, including axillary bud induction and *in vitro* plantlet (**Figure 5**). GFP expression was observed in both the axillary buds and leaves of transgenic plantlets (**Figure 5**). An advantage of visualizing GFP expression in our system was to enable us to select transformation events at an early stage thus avoiding the transfer of non-transgenic shoot that survived the kanamycin selection, saving both time and labor.

### MOLECULAR ANALYSIS OF TRANSGENIC PLANTS

**FIGURE 6:**
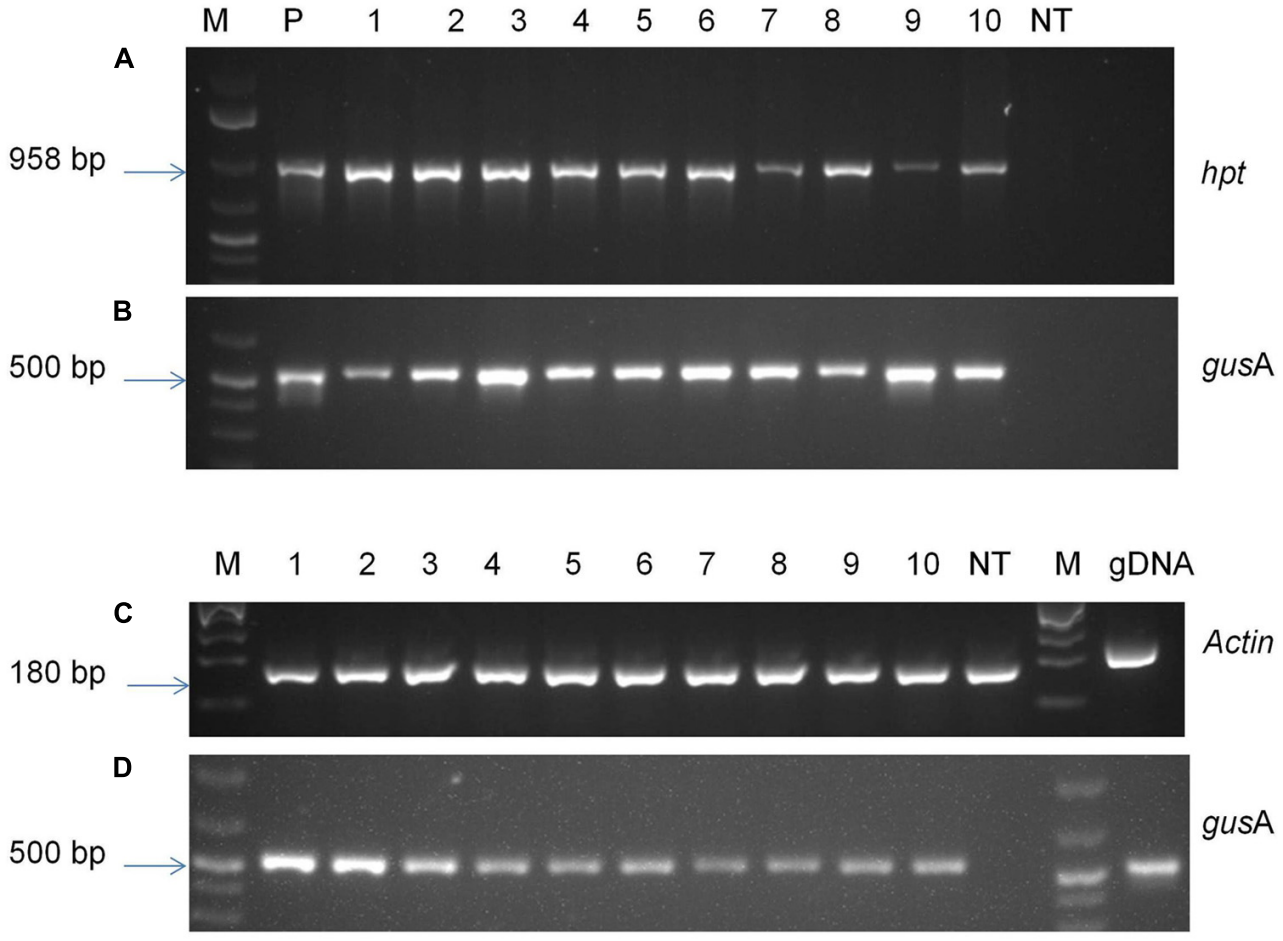
**Molecular analysis of transgenic plants.** PCR analysis of genomic DNA of putative transgenic and non-transgenic control plants using primers specific for **(A)** *hpt* gene; **(B)** *gusA* gene; RT-PCR analysis using primers specific to **(C)** *Actin* gene; **(D)** *gusA* gene. M- 1 kb plus molecular marker (Fermentas); P- pCAMBIA1301 plasmid DNA; 1–10- transgenic plants; NT- control non-transgenic plant.

To confirm the presence of foreign genes into the genome of transgenic plants, antibiotic-resistant plants were analyzed by PCR and RT-PCR (**Figure 6**). PCR analysis was performed with genomic DNA of putative transgenic and control non-transgenic plants in order to confirm the presence of transgene. The amplified product of about 500 base pairs corresponding to the internal fragment of *gus*A gene was observed from genomic DNA of all the transgenic plants tested using *gus*A gene-specific primers confirming the presence *gus*A transgene in transgenic plants. An amplified fragment of 958 base pairs was also observed from all tested transgenic plants using *hpt* specific primers confirming the presence of *hpt* gene. The amplified products were observed in all the plants tested, confirming the presence of both transgenes *gus*A and *hpt*, without any escape plant. No amplified product was observed in case of non-transgenic control plants.

The transgenic lines were analyzed using RT-PCR in order to verify the expression of *gus*A gene. The *gus*A transcript amplification of the expected fragment size (∼500 base pair) was observed from samples of all the transgenic lines tested (**Figure 6**). Specific *Actin* transcript amplification was detected from all plants as an internal control for cDNA synthesis. A gDNA control was included in the assay with actin primers and showed the larger unspliced fragments, indicating that DNA contamination was below PCR detection levels in RNA samples. The results indicated that target genes were successfully incorporated into plant genome and were expressed in transgenic plants.

**FIGURE 7:**
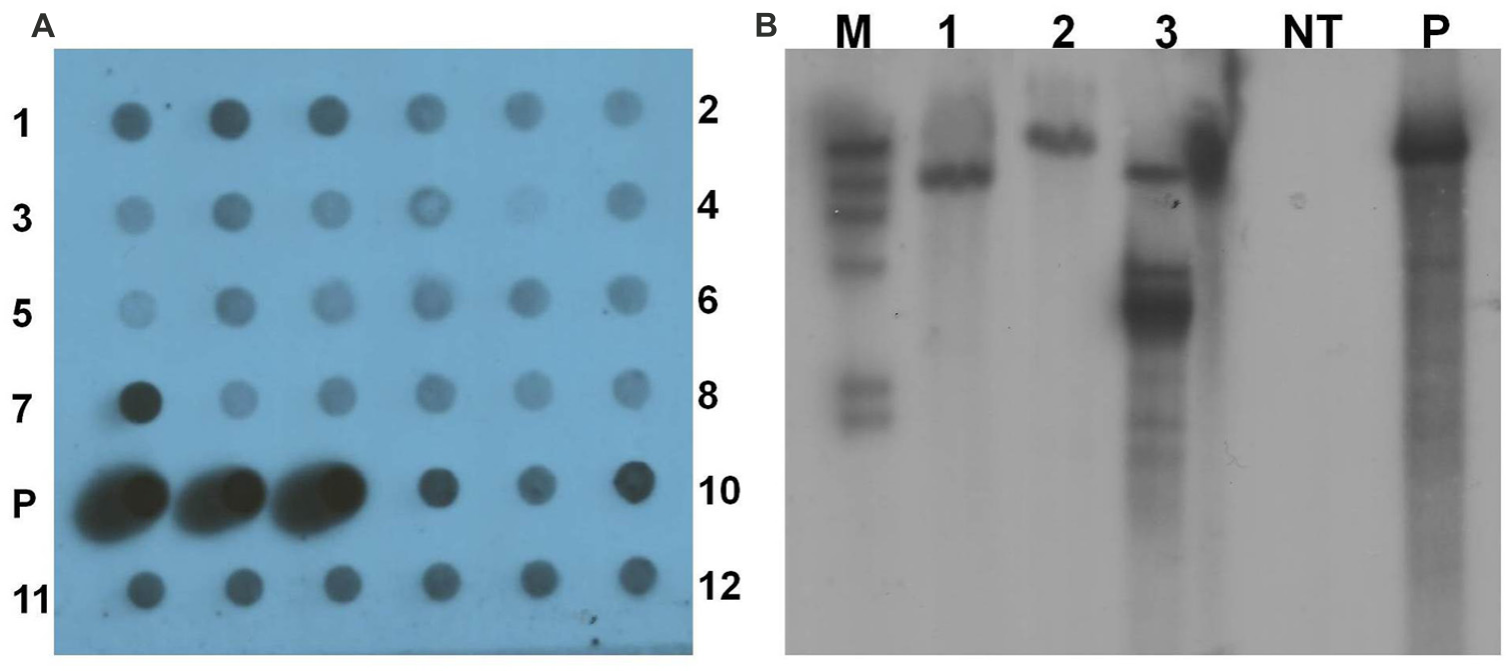
**Analysis of transgenic lines to confirm integration of transgene. (A)** Dot blot analysis of transgenic lines. 1–12- transgenic lines in triplicates; P- pCAMBIA1301 plasmid DNA as positive control. **(B)** Southern blot analysis of genomic DNA of transgenic lines and non-transgenic control plant digested with *Hind*III. M- DIG-labeled molecular weight marker; 1–3- transgenic lines; NT- non-transgenic plant; and P- pCAMBIA1301 plasmid DNA as positive control.

Polymerase chain reaction positive transgenic lines were further analyzed by dot blot and Southern blot hybridization using *gusA* probe to confirm integration of the transgene into the genome of yam. Genomic DNA of all the 12 transgenic lines tested by dot blot analysis were confirmed to contain the *gus*A gene (**Figure 7A**). Three transgenic lines were further tested with Southern blot hybridization. Their unique hybridization patterns indicated that each transgenic line resulted from an independent transformation event (**Figure 7B**). No hybridization signal was detected in the non-transgenic control plant.

### FUTURE PROSPECTS OF GENETIC ENGINEERING FOR YAM IMPROVEMENT

Pests and diseases are among the most important of the many factors that have deleterious effects on yam tuber yield and quality and over time these constraints have become more severe (Amusa et al., 2003; Baimey et al., 2006; Aidoo et al., 2011). The progress made in this study in establishing an efficient genetic transformation system of *D. rotundata* could open up many avenues to produce disease resistant yams, through pathogen-derived resistance strategies, that would not be possible using conventional breeding approaches. Host plant resistance to anthracnose has been proposed as a viable alternative to the use of chemical fungicides in controlling the disease. However, studies have shown that there are no genotypes tolerant or resistant to the disease (Abang et al., 2002). Therefore, the most attractive strategy for anthracnose control in yam is probably the production of disease resistant plants through the transgenic approach. These approaches could include the expression of genes encoding plant, fungal or bacterial hydrolytic enzymes (Lorito et al., 1998), genes encoding elicitors of defense response (Keller et al., 1999) and antimicrobial peptides (AMPs; Broekaert et al., 1997). AMPs have a broad-spectrum antimicrobial activity against fungi as well as bacteria and most are non-toxic to plant and mammalian cells.

Use of resistant varieties can be an effective strategy in controlling yam nematodes, but there are no varieties known to be tolerant to nematodes. The use of transgenic plants will be an alternative approach to improve the nematode resistance of yam. Several transgenes confer plant resistance to both tropical and temperate plant parasitic nematodes (Atkinson et al., 2003). Cystatins inhibit nematode digestive cysteine proteinase activity, suppressing the growth and multiplication of these pests (Urwin et al., 1997) and is one of the transgenes that has been successfully used to control plant nematodes. It has been found that the cystatins confers the improved resistance to a range of nematodes in different crops like potato, sweet potato, rice, tomato, and plantain (Atkinson et al., 1996; Vain et al., 1998; Urwin et al., 2001; Chan et al., 2010; Gao et al., 2011; Roderick et al., 2012) and have proven efficacy under field conditions (Urwin et al., 2001, 2003). Such an approach could also be used to enhance resistance of yam against nematodes in the near future.

## CONCLUSION

We have established a highly efficient and simple *Agrobacterium*-mediated transformation protocol for *D. rotundata* using axillary buds as explants. Stable transgenic plantlets which showed presence, integration, and expression of transgenes were successfully regenerated within 3–4 months from axillary bud explants. To the best of our knowledge, this is the first report of *Agrobacterium*-mediated transformation of yam with experimental evidence of stable integration of T-DNA in *D. rotundata* genotypes. This protocol opens up an avenue for future genetic improvement of *D. rotundata* with candidate genes of proven agronomic importance to attain sustainable production.

## Conflict of Interest Statement

The authors declare that the research was conducted in the absence of any commercial or financial relationships that could be construed as a potential conflict of interest.
